# A comparison study of succinct data structures for use in GWAS

**DOI:** 10.1186/1471-2105-14-369

**Published:** 2013-12-21

**Authors:** Patrick P Putnam, Ge Zhang, Philip A Wilsey

**Affiliations:** 1 Experimental Computing Lab, School of Electronic and Computing Systems, PO Box 210030, Cincinnati, OH 45221–0030, USA; 2 Human Genetics, Cincinnati Children’s Hospital Medical Center, Cincinnati, OH, USA

## Abstract

**Background:**

In recent years genetic data analysis has seen a rapid increase in the scale of data to be analyzed. Schadt *et al* (NRG 11:647–657, 2010) offered that with data sets approaching the petabyte scale, data related challenges such as formatting, management, and transfer are increasingly important topics which need to be addressed. The use of succinct data structures is one method of reducing physical size of a data set without the use of expensive compression techniques. In this work, we consider the use of 2- and 3-bit encoding schemes for genotype data. We compare the computational performance of allele or genotype counting algorithms utilizing genotype data encoded in both schemes.

**Results:**

We perform a comparison of 2- and 3-bit genotype encoding schemes for use in genotype counting algorithms. We find that there is a 20% overhead when building simple frequency tables from 2-bit encoded genotypes. However, building pairwise count tables for genome-wide epistasis is 1.0% more efficient.

**Conclusions:**

In this work, we were concerned with comparing the performance benefits and disadvantages of using more densely packed genotype data representations in Genome Wide Associations Studies (GWAS). We implemented a 2-bit encoding for genotype data, and compared it against a more commonly used 3-bit encoding scheme. We also developed a C++ library, libgwaspp, which offers these data structures, and implementations of several common GWAS algorithms. In general, the 2-bit encoding consumes less memory, and is slightly more efficient in some algorithms than the 3-bit encoding.

## Background

In recent years genetic data analysis has seen a rapid increase in the scale of data to be analyzed. Schadt *et al*[1] offered that with data sets approaching the petabyte scale, data related challenges such as formatting, management, and transfer are increasingly important topics which need to be addressed.

The majority of tools used in GWA data analysis typically assume that a data set will easily fit into the main memory of a desktop computer. Most desktop computers have around 4–16 GB of main memory, which is more than enough to fit a data set of 1 million variants by tens of thousands of individuals. However, data set sizes continue to grow with advancements in analysis techniques and technologies. For example, techniques like genotype imputation [2] attempt expand data sets by deriving missing genotype from reference panels. Genotyping technologies such as Illumina’s Omni SNP HumanOmni5-Quad chips allow for genotyping of upwards of 5 million markers [3]. Furthermore, genome sequencing technologies are advancing to the point where determining genotypes via whole genome sequencing may be a viable option. Having an individual’s entire DNA sequence opens the door for even more genetic markers to be analyzed. The 1000 Genomes project [4] now includes roughly 36.7 million variants in the human genome.

The size of a data file used to represent the genotypes of 1000 individuals would be roughly 37 GB (assuming 1 byte is used to store each genotype). There are a several options to handling data sets of this size. First, the cost of upgrading a standard PC’s memory to handle this amount of data is not unreasonable. Second, the algorithm can be extended to utilize memory mapping techniques [5], which effectively pages chunks of the data file into main memory as they are needed. A third option is to modify the format for representing genotypes such that the genotypes are expressed in their most succinct form [6,7]. This manuscript explores the latter option more deeply. The interest is motivated in part by the desire to work in the General-Purpose Graphic Processing Units (GPGPU) space which has somewhat limited space especially when considered on a processor-by-processor basis.

The compression of genotype encoding data is most effectively performed using succinct data structures [8]. Succinct data structures allow compression rates close to the information-theoretic limits and yet preserve the ability to access individual data elements. In the genotype analysis tools that use succinct data types (*e.g.*, BOOST [6] and BiForce [9]), a 3-bit genotype representation for biallelic markers has been adopted. While a 3-bit representation does provide a succinct data structure, it is not the most succinct. More precisely, from an information theoretic perspective, 3-bits is able to represent up to 8 unique values. However, there are only 4 commonly used unphased genotypes, namely {NN, AA, Aa, aa} where NN is used to represent missing data. This means that a 2-bit representation is the information theoretic lower bound and its use would provide an even more compact representation.

An important consideration when designing succinct data structures is data element orientation in memory. BOOST [6] and BiForce [9] adopted a vectored orientation for representing data elements. The vectored orientation spreads each data element over multiple bit vectors. In other words, they utilize 3 bit vectors per marker to represent the set of genotypes. The advantages of this orientation are discussed later.

This manuscript makes two important contributions in the use of succinct data structures for genomic encoding. In particular, (i) we implement a technique to reduce genotype encoding to a 2 bit vector form, and (ii) we compare the performance of the new 2-bit encoding to the conventional 3 bit vector encoding. From these studies, we have observed that the 2-bit encoding encoding consumes less memory, and is slightly more efficient in some algorithms than the 3-bit encoding.

## Implementation

We analyzed a commonly used 3-bit binary representation of genotypes from performance and scalability perspectives. With this information we developed a C++ object library that we have named libgwaspp. The library provides data structures for managing genotype data tables in a 2- or 3-bit representation. Finally, we benchmarked the two representations on randomly generated data sets of various scales.

### Genome-wide association studies

DNA from individuals are collected, sequenced or genotyped, and the genotypes for genetic variants are used in Genome-Wide Association Studies (GWAS). These studies aim to determine whether genetic variants are associated with certain traits, or phenotypes. The most common studies are case-control studies which group individuals together into two sets based on the presence (case) or absence (control) of a specific trait. These studies typically rely upon various statistical tests based upon the genotypic or allelic distribution of the variants in each set. An average data set aims to compare thousands of individuals by hundreds of thousands to millions of variants.

GWA studies can be computationally intensive to perform. Common algorithms consider either each variant individually, or variants in combination with one another. For example, measuring the odds ratio for each variant in a case-control study is one way of identifying variants which may be associated with the trait in question. An epistasis analysis algorithm, such as BOOST [6], compares the genotype distribution of two variants in each step.

In both of these algorithms, the basic task is counting the occurrences of each genotype in each of the case-control sets. In other words, the first step in determining the odds ratio is to build a frequency table (Table 1) for both the case and control sets at a specific variant. Similarly, the BOOST [6] algorithm first builds a contingency table (Table 2), or pairwise genotype count table, for a pair of variants.

**Table 1:** **Frequency table for raw input from Tables **3, 4 **and**5

	**AA**	**Aa**	**aa**	**NN**
*C*_*A*_	2	1	1	1
*C*_*B*_	2	1	2	0

**Table 2:** Pairwise genotype count table for two markers

		***M***_***B***_				
		**AA**	**Aa**	**aa**	**NN**	***C***_***A***_
*M*_*A*_	AA	1	0	1	0	2
	Aa	1	0	0	0	1
	aa	0	0	1	0	1
	NN	0	1	0	0	1
	*C*_*B*_	2	1	2	0	

Note that the marginal sums of this table are the individual markers frequencies from Table 1.

**Table 3:** Example genotype input

	***I***_**1**_	***I***_**2**_	***I***_**3**_	***I***_**4**_	***I***_**5**_
*M*_A_	AA	Aa	AA	aa	NN
*M*_B_	AA	AA	aa	aa	Aa

*I*_1-5_ represent individuals, and *M*_A_ and *M*_B_ are markers.

**Table 4:** 3-bit encoding scheme

		***I***_**1**_	***I***_**2**_	***I***_**3**_	***I***_**4**_	***I***_**5**_
	AA	1	0	1	0	0
*M*_A_	Aa	0	1	0	0	0
	aa	0	0	0	1	0
	AA	1	1	0	0	0
*M*_B_	Aa	0	0	0	0	1
	aa	0	0	1	1	0

**Table 5:** 2-bit encoding scheme

		***I***_**1**_	***I***_**2**_	***I***_**3**_	***I***_**4**_	***I***_**5**_
*M*_A_	AA OR aa	1	0	1	1	0
	Aa OR aa	0	1	0	1	0
*M*_B_	AA OR aa	1	1	1	1	0
	Aa OR aa	0	0	1	1	1

### Binary genotype encoding schemes

A common way to minimize the impact of the table building bottleneck is to fully utilize processor throughput by counting genotypes from multiple individuals in one step. The binary encoding of genotypes adopted by BOOST [6] improves the computational efficiency of the epistasis algorithm. The algorithm used 3 bit vectors to encode for genotype data. In this scheme each genotype is its own bit-vector, or stream, of data. Each bit corresponds to an indexed individual, and the indexing is assumed to be constant across all markers. A set bit indicates that the individual has the corresponding genotype for the specified marker. Therefore, every variant requires 3 vectors to fully represent the genotypes.

There are two key benefits of using this binary encoding scheme. The first is that the task of building a frequency table for a given marker is reduced to calculating the Hamming distance of each of a bit-vectors and a bit-vector of all zeros. This distance is also referred to as a Hamming weight. The technique used for calculating the Hamming weight of a bit vector is to divide the bit-vector into manageable blocks, and sum the Hamming weight of each block. The block size is typically linked to the processor word size, typically 32- or 64- bits (4 or 8 bytes). The algorithm for computing the Hamming weight of an individual block is commonly referred to as Population Counting (popcount). We chose to follow the BOOST implementation of popcount which looks-up the Hamming weight of 16-bit blocks in a pre-populated weight table. The second benefit is that it reduces genotype comparison logic to simple Boolean logic operations. More specifically, the task of counting individuals which have a specific combination of genotypes for two markers is simplified to finding the Hamming weight of the logical AND of the genotype bit vectors. This is useful when building contingency tables.

Of interest to this paper is the fact that when using the 3-bit encoding scheme at least two thirds of the bits used will be unset. An information theoretic analysis of the genotype alphabet indicates that 2-bits are sufficient to uniquely represent each of the four unphased genotypes. The immediate benefit is a one third reduction in memory consumption (Tables 3, 4 and 5). The caveat to this encoding scheme is that determining a genotype requires both bits.

**Figure 1:**
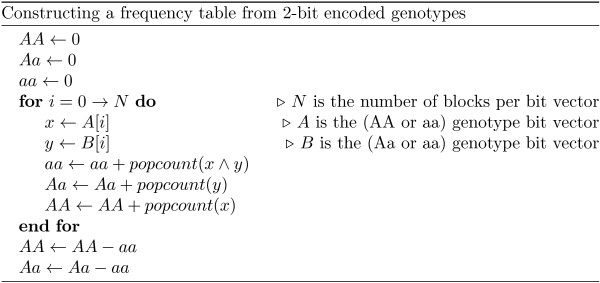
Constructing a frequency table from 2-bit encoded genotypes.

The algorithm in Figure 1 is a pseudo-code representation of how to build a genotype count table from 2-bit encoded data. The Hamming weight of each vector is the number of individuals with (AA or aa), and (Aa or aa) genotypes, respectively. To disambiguate the values it is necessary to compute the Hamming weight of the logical AND of the bit-vectors. This value represents the number of (aa) genotypes, and subtracting it from the previous two weights will result in the appropriate counts.

**Figure 2:**
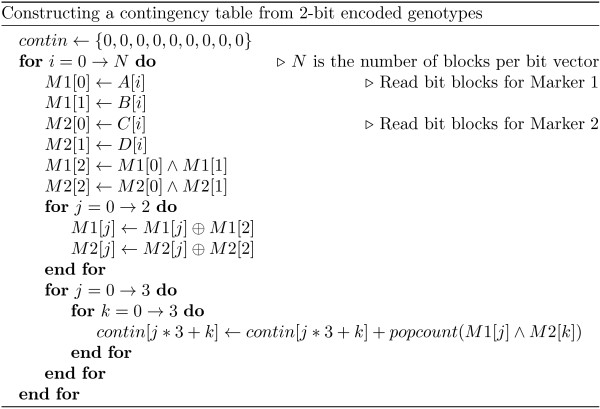
Constructing a contingency table from 2-bit encoded genotypes.

The algorithm in Figure 2 illustrates the construction of a pairwise genotype count table, or contingency table. A contingency table represents the number of individuals who possess a genotype combination for a pair of markers. When using the 3-bit encoding scheme, each cell of the table is simply the Hamming weight of the logical AND of the genotype bit-vectors for the two markers. The 2-bit encoding requires an inline transformation step to convert the 2-bit encoded data into 3-bit data. This step is necessary to be able to take advantage of the popcount bit counting method.

Both of the above algorithms can be further improved by incorporating additional information. For example, the algorithm for building a contingency table can be simplified if marginal information for both variants is available. The contingency table algorithm can make use of the variants’ frequency table and reduce having to compute 9 Hamming weight values to only 4. The remaining values can be easily computed by subtracting the row and column sums from their respective marginal information values. This reduction offers significant computational savings, especially when performing exhaustive epistasis analysis.

### Benchmarking

We compared the performance of the 2-bit encoded data to the 3-bit encoded data. In particular, we measured the runtime for building frequency tables and contingency tables using both encoding schemes. The runtime of these algorithms are dependent upon the number of columns, or individuals, in each row. Therefore, we decided to hold the number of rows constant at 10,000 variants. We varied the number of columns between 1 and 50 thousand individuals. We also tested a set with 150,000 individuals as an extreme scale experiment. The genotypes were simulated following empirical allele frequency spectrum of Affymetrix array 6.0 SNPs of the CEU HapMap samples. Similarly, individuals were randomly classified as either a case or control.

Three experiments were conducted. First, for each data set the runtime for building frequency tables for each of the variants were measured. Second, for each data set the runtime for building all contingency tables for an exhaustive pairwise epistasis test was measured. Third, each data set was run through our implementation of the BOOST [6] algorithm and the total runtime was recorded. The runtime of BOOST [6] algorithm does not include the time to load the compressed data set into main memory. In each of these tests, the average runtime is calculated and presented.

All tests were conducted upon a desktop computer with an 3.2 GHz Intel Core i7-3930K, 32 GB of 1600 MHz DDR3 memory, with 64-bit Fedora 17. Time was measured down to the nanosecond using the clock_gettime() glibc function. We used GNU G++ compiler 4.7, and compiled using standard “-O3” compiler optimization flag. The tests were performed using 64-bit block size.

## Results

**Figure 3:**
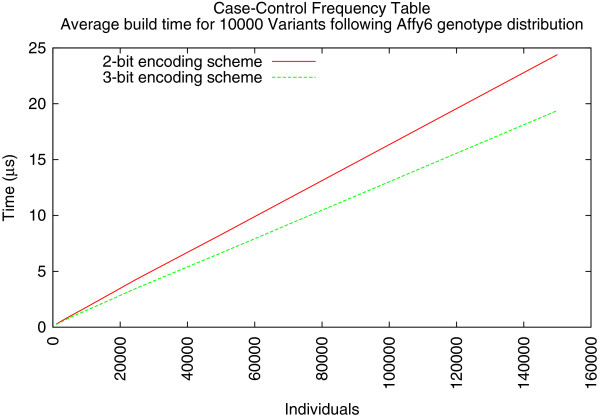
Average Case/Control frequency table construction using simulated data following Affy6 SNPs of HapMap CEU individuals.

The first experiment measured the runtime for building frequency tables. Initially, the 3-bit encoding scheme appeared to offer a consistent performance advantage over the 2-bit encoding. As the number of individuals increased, it took less time to construct the count table (Figure 3). The average time to build a genotype count table for less than 10,000 individuals is less than 1 *μs*. For data sets greater than 10,000 individuals, there is some performance overhead that results from decoding the 2-bit vectors. Building frequency tables from the 3-bit encoded data proved to be 12–25% faster than when built from 2-bit encoded data. In the extreme scale data set there was a 5.00 *μs* difference in favor of the 3-bit scheme. However, the second experiment offered different results.

**Figure 4:**
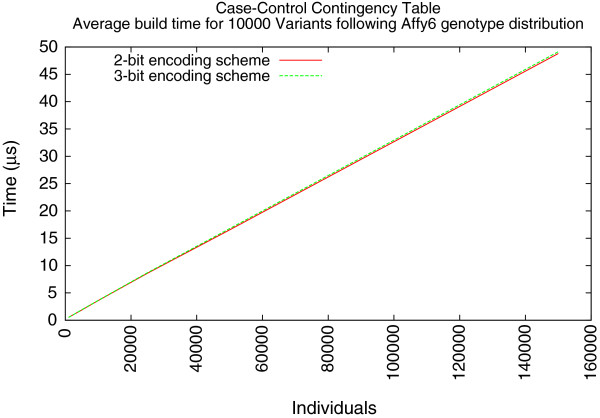
Average Case/Control contingency table construction using simulated data following Affy6 SNPs of HapMap CEU individuals.

The second experiment measured the runtime for building contingency tables for all pairs of variants in the data sets. In this experiment, the 2-bit encoding scheme offered better performance. Similar to the first experiment, 10,000 individuals seemed to be the diverging point (Figure 4). At sizes greater than 10,000 individuals, the 2-bit encoding scheme offered a 1% performance improvement over the 3-bit scheme. With 150,000 individuals, this equates to about a 0.32 *μs* difference in average performance. The third experiment further confirms this performance gain (Table 6).

**Table 6:** Epistasis runtime comparison

**Individuals**	**2-bit**	**3-bit**	**Speedup(%)**
1000	28.56 s	28.45 s	0.37
5000	92.07 s	93.32 s	-1.33
10000	173.12 s	177.46 s	-2.45
25000	418.31 s	420.71 s	-0.57
50000	810.71 s	820.26 s	-1.16
150000	2408.05 s	24.27.84 s	-0.81

Speedup is measured relative to the 3-bit runtime.

## Discussion

This work focuses on ways to address frequency table building processes found in GWAS for two primary reasons. First, upstream steps, like the loading of data, in a general GWAS pipeline are performed relatively infrequently, and can be performed offline. For example, a data set can be transformed into an optimized format once, and in every repeat analysis the data set the loading becomes a constant time step within the pipeline. Conversely, the building of these tables amounts to a frequently reoccurring step which is typically performed inline under varying conditions.

Secondly, we viewed the table building process as a bottleneck for downstream analytical steps. Offering an approach which positively impacts the cost associated with this bottleneck is beneficial.

The results suggest that the use of 2-bit encoding scheme for genotype data does offer several benefits over a 3-bit encoding scheme. The compact encoding scheme requires 33% less memory for representing the same data. Aside from freeing up system memory for other tasks, the memory savings can be beneficial for other reasons. For example, epistasis algorithms like BOOST [6] can be run on Graphic Processing Units. GPUs are separate devices on a computer which have their own physical memory, typically less than 6 GB, and require data to be copied to and from the device. The limited memory and data transfer issues both benefit from using a more compact data format.

The 2-bit encoded genotypes have also been used by other software packages. PLINK [7], for example, uses a 2-bit encoding in the BED file format. BED files use a contiguous pairing of bits to express the genotype of an individual. Using bit pairs allows for more efficient individual genotype decoding as a result of the bits existing in the same bit-block. However, additional bit masking steps need to be applied to each block to effectively utilize popcount based methods for counting genotype occurrences within a block. As mentioned earlier, our implementation adopts a bit-vectored approach, whereby an individual’s genotype is divided over two separate vectors. This is primarily done to reduce the number of masking steps.

In either case, some form of genotype disambiguation is necessary. There is an overhead associated with this decoding step, and it can be felt in certain algorithms. We measured approximately a 20% overhead when building frequency tables. While this is a significant overhead, the number of frequency tables are linear in the number of markers. Therefore, it is conceivable to build these tables once, and reuse them in downstream analytical steps as needed. As a result, this overhead is generally acceptable. Furthermore, the overhead is effectively hidden when building pairwise frequency tables.

The improvement in performance present when constructing pairwise frequency tables from 2-bit encoded genotypes stems from the reduced number of memory access steps. As shown in Algorithm 3 six genotypes blocks are used in each step of the iteration. When 3-bit encoding is used, each of these blocks must be read from memory. Conversely, the 2-bit encoding only needs to read four blocks and computes the remaining two blocks.

A further general performance increase may be possible through the use of hardware implementations of popcount algorithms. As part of the Streaming SIMD Extensions (SSE) of the x86 microarchitecture there is a popcnt [10] instruction. Recent processor lines from both Intel and AMD offer this instruction in some form or another.

**Figure 5:**
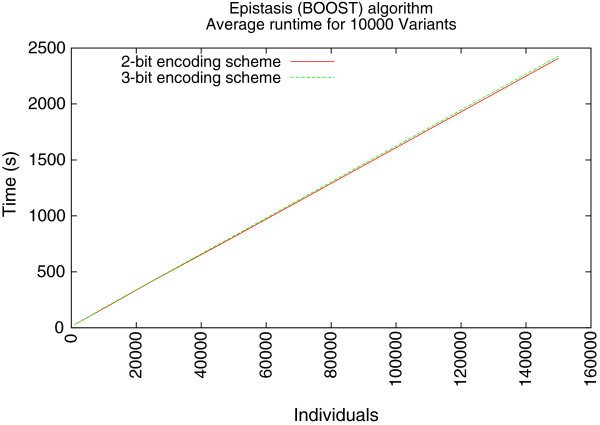
**Average epistasis runtime using BOOST **[6]** algorithm.**

As we mentioned earlier, these succinct data structures are intended to impact the increasing scale of sample sets. The building of the frequency tables are linear algorithms which are dependent upon the sample sets. By fixing the number of variants and varying the number of samples in a data set we show the linear increase of the epistasis algorithm runtime, as is indicated by Figure 5.

Unfortunately, the runtime of brute force algorithms like BOOST [6] are dominated more by the number of variants being analyzed than the number of individuals being studied. A data set of 10,000 variants means that 5×10^7^ unique contingency tables need to be built for a typical case-control study. Expanding that size to a million variants increases the contingency table count to 5×10^11^. Other works have demonstrated parallel implementations that effectively address the variant scaling [9]**,**[11]**,**[12]. This work demonstrates a general way to further improve the performance of these algorithms.

## Conclusions

In this work, we were concerned with comparing the performance benefits and disadvantages of using more densely packed data representations in Genome Wide Associations Studies. We implemented a 2-bit encoding for genotype data, and compared it against a more commonly used 3-bit encoding scheme. We also developed a C++ library, libgwaspp, which offers these data structures, and implementations of several common GWAS algorithms. In general, the 2-bit encoding consumes less memory, and is slightly more efficient in some algorithms than the 3-bit encoding.

## Availability and requirements

**Project name:** libgwaspp

**Project home page:**https://github.com/putnampp/libgwaspp

**Operating system(s):** Linux

**Programming language:** C++

**Other requirements:** CMake 2.8.9, GCC 4.7 or higher, Boost 1.51.0, ZLIB, GSL

**License:** FreeBSD

## Competing interests

The authors declare that they have no competing interests.

## Authors’ contributions

PPP designed and implemented the software, conducted the experiments, and wrote the main manuscript. GZ provided domain specific expertise in GWA studies, and the empirical data from which the simulated data was generated. PW contributed extensive knowledge of computational architectures and data structures. Both also contributed greatly to the result analysis and editing of the manuscript. All authors read and approved the final manuscript.

## References

[B1] Schadt EE Linderman MD Sorenson J Lee L Nolan GP Computational solutions to large-scale data management and analysis Nat Rev Genet 2010 14 9 647 657 http://dx.doi.org/10.1038/nrg2857 10.1038/nrg2857 20717155 PMC3124937

[B2] Li Y Willer C Sanna S Abecasis G Genotype imputation Ann Rev Genom Human Genet 2009 14 387 406 http://www.annualreviews.org/doi/abs/10.1146/annurev.genom.9.081307.164242. [PMID: 19715440]. 10.1146/annurev.genom.9.081307.164242 PMC2925172 19715440

[B3] Whole-genome genotyping and copy number variation analysis 2013 http://www.illumina.com/applications/detail/snp_genotyping_and_cnv_analysis/whole_genome_genotyping_and_copy_number_variation_analysis.ilmn. [Online; accessed 9-January-2013]

[B4] A map of human genome variation from population-scale sequencing Nature 2010 14 7319 1061 1073 http://dx.doi.org/10.1038/nature09534 10.1038/nature09534 20981092 PMC3042601

[B5] Nielsen J Mailund T SNPFile - A software library and file format for large scale association mapping and population genetics studies BMC Bioinformatics 2008 14 526 http://www.biomedcentral.com/1471-2105/9/526 10.1186/1471-2105-9-526 19063732 PMC2633306

[B6] Wan X Yang C Yang Q Xue H Fan X Tang NL Yu W BOOST: a fast approach to detecting gene-gene interactions in genome-wide case-control studies Am J Human Genet 2010 14 3 325 340 http://linkinghub.elsevier.com/retrieve/pii/S0002929710003782 10.1016/j.ajhg.2010.07.021 20817139 PMC2933337

[B7] Purcell S Neale B Todd-Brown K Thomas L Ferreira MAR Bender D Maller J Sklar P de Bakker PIW Daly MJ Sham PC PLINK: a toolset for whole-genome association and population-based linkage analysis Am J Human Genet 2007 14 3 559 575 http://pngu.mgh.harvard.edu/purcell/plink/10.1086/519795 17701901 PMC1950838

[B8] Jacobson G Space-efficient static trees and graphs Proceedings of the 30th Annual Symposium on Foundations of Computer Science, SFCS ’89 1989 Washington: IEEE Comput Soc 549 554 http://dx.doi.org/10.1109/SFCS.1989.63533

[B9] Gyenesei A Moody J Laiho A Semple CA Haley CS Wei WH BiForce Toolbox: powerful high-throughput computational analysis of gene-gene interactions in genome-wide association studies Nucleic Acids Res 2012 14 W1 W628 W632 http://nar.oxfordjournals.org/content/40/W1/W628.abstract 10.1093/nar/gks550 22689639 PMC3394281

[B10] Intel Intel SSE4 Programming Reference 2007. http://home.ustc.edu.cn/~shengjie/REFERENCE/sse4_instruction_set.pdf

[B11] Yung LS Yang C Wan X Yu W GBOOST: a GPU-based tool for detecting geneŰgene interactions in genome-wide case control studies Bioinformatics 2011 14 9 1309 1310 http://bioinformatics.oxfordjournals.org/content/27/9/1309.abstract 10.1093/bioinformatics/btr114 21372087 PMC3105448

[B12] Schüpbach T Xenarios I Bergmann S Kapur K FastEpistasis: a high performance computing solution for quantitative trait epistasis Bioinformatics 2010 14 11 1468 1469 http://bioinformatics.oxfordjournals.org/content/26/11/1468.abstract 10.1093/bioinformatics/btq147 20375113 PMC2872003

